# Enhanced cellular uptake of lactosomes using cell-penetrating peptides

**DOI:** 10.1080/14686996.2016.1178056

**Published:** 2016-06-08

**Authors:** Akiya Akahoshi, Eiji Matsuura, Eiichi Ozeki, Hayato Matsui, Kazunori Watanabe, Takashi Ohtsuki

**Affiliations:** ^a^ Department of Medical Bioengineering, Okayama University, Okayama700-8530, Japan; ^b^ Collaborative Research Center for OMIC, Okayama University, Okayama700-8558, Japan; ^c^ Technology Research Laboratory, Shimadzu Corporation, Kyoto, Japan

**Keywords:** Cell penetrating peptide, polymeric micelle, drug delivery, photosensitizer, lactosome, 30 Bio-inspired and biomedical materials, 211 Scaffold/Tissue engineering/Drug delivery, 200 Applications

## Abstract

Polymeric micelles that are composed of synthetic polymers are generally size controllable and can be easily modified for various applications. Lactosomes (A_3_B-type) are biodegradable polymeric micelles composed of an amphipathic polymer, including three poly(sarcosine) blocks and a poly(l-lactic acid) block. Lactosomes accumulate in tumors *in vivo* through the enhanced permeability and retention (EPR) effect, even on frequently administering them. However, lactosomes cannot be efficiently internalized by cells. To improve cellular uptake of lactosomes, cell-penetrating peptide (CPP)-modified lactosomes were prepared. Seven CPPs (including EB1 and Pep1) were used, and most of them improved the cellular uptake efficiency of lactosomes. In particular, EB1- and Pep1-modified lactosomes were efficiently internalized by cells. In addition, by using CPP-modified and photosensitizer-loaded lactosomes, we demonstrated the photoinduced killing of mammalian cells, including human cancer cells. Accumulation of the EB1-modified lactosomes in NCI-N87 tumors was shown by *in vivo* imaging. Thus, this study demonstrated that the CPP-modified lactosome is a promising drug carrier.

## Graphical abstract

**Figure:**
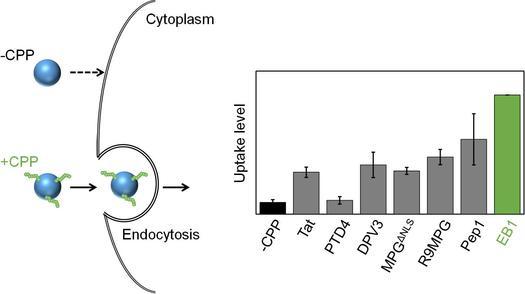

## 1. Introduction

The use of nanoparticles as drug carriers for the selective delivery of pharmacological agents in various diseases such as cancer and viral infections has been studied.[1,2] Over the past few decades, many nanoparticles, including liposomes,[3] polymeric micelles,[4,5] metal nanoparticles,[6] and dendrimers,[7] have been developed for drug delivery. Among them, polymeric micelles are size-controllable and easily modified for adding new abilities, such as targeting and imaging. The polymers that comprise the micelles are size-controllable and can have various reactive groups. Several liposome drugs have been approved and are in clinical use, and polymeric micelles are in the clinical test phase.[5,8,9]

Lactosomes are polymeric micelles composed of an amphipathic polymer with a hydrophilic poly(sarcosine) (PSar) block and a hydrophobic poly(l-lactic acid) (PLLA) block (AB-type).[10,11] The high density of PSar chains around the molecular assemblies contributes to their ability to escape from the reticuloendothelial system (RES) recognition, similarly to polyethylene glycol (PEG) modification.[12] Furthermore, PSar is considered to have an advantage over PEG in terms of biodegradability. AB-type lactosomes persist in the bloodstream for a long time.[13] However, AB-type lactosomes exhibit a drawback in multiple *in vivo* administrations,[14] in which they accumulate in solid tumors after the first administration, but accumulate only slightly after the second administration. To compensate for this drawback, a lactosome composed of (PSar)_3_-block-PLLA (A_3_B-type) has recently been developed.[15] The AB-type and A_3_B-type lactosomes accumulate in tumor tissue through the enhanced permeation and retention (EPR) effect.[16] However, the lactosomes are not efficiently internalized by cells even if they accumulate around them. Improving the cellular uptake of the lactosomes is important for the efficient delivery of drugs that act intracellularly.

In this study, we attempted to modify A_3_B-type lactosomes with cell-penetrating peptides (CPPs) to increase efficiency of their cellular uptake. CPPs are short peptides often rich in cationic residues, which have the ability to internalize various cargo molecules such as nucleic acids, proteins, and nanoparticles.[17–19] We used seven CPPs, including those derived from natural proteins (Tat and DPV3) [20–22] and chimeric or artificial peptides (PTD4, MPG^ΔNLS^, Pep1, and EB1).[23–26] Cellular uptake efficiency of various CPP-modified lactosomes was compared. In addition, the photosensitizer 5,10,15,20-tetraphenyl-21*H*,23*H*-porphyrin (TPP) [27] was delivered to mammalian cells (including cancer cell lines) by the CPP-modified lactosomes. The cells were treated with the TPP-encapsulated lactosome and photoirradiated to evaluate the photodynamic therapy (PDT) effect. Finally, *in vivo* localization of the CPP-modified lactosomes was investigated using tumor-bearing mice.

## 2. Experimental details

### 2.1. Materials

(PSar_25_)_3_-block-PLLA_35_, indocyanine green (ICG)-PLLA_34_, and maleimide-PSar_56_-PLLA_30_ were synthesized as described.[11,15,28] Chinese hamster ovary (CHO) cells (FLIP-In cell line) were purchased from Invitrogen (Waltham, Massachusetts, USA). NCI-N87 human gastric cancer cells and PANC-1 human pancreatic carcinoma cells were purchased from the American Type Culture Collection. The CPPs listed in Table 1 were prepared by Fmoc-based solid-phase peptide synthesis and provided by the Central Research Laboratory at the Okayama University Medical School.

**Table 1.** Cell-penetrating peptides used in this study.

CPP	Classification	Length	Sequence
Tat	Cationic	11	YGRKKRRQRRR-C
PTD4	Cationic	11	YARAAARQARA-C
DPV3	Cationic	16	RKKRRRESRKKRRRES-C
MPG^ΔNLS^	Amphipathic	27	GALFLGFLGAAGSTMGAWSQPKSKRKV-C
R9MPG	Amphipathic	25	RRRRRRRRRGALFLAFLAAALSLMG-C
Pep1	Amphipathic	21	KETWWETWWTEWSQPKKKRKV-C
EB1	Amphipathic	23	LIRLWSHLIHIWFQNRRLKWKKK-C

### 2.2. Preparation of lactosome complexes

A chloroform solution containing the (PSar_25_)_3_-block-PLLA_35_ (100 nmol, 7.7 μg) with 4 mol% of TPP (Wako Pure Chemical, Osaka, Japan), 2 mol% of ICG-PLLA_34_, and 10 mol% maleimide-PSar_56_-PLLA_30_ was evaporated under reduced pressure to remove the solvent, forming a thin film on the surface of the glass test tube. Saline (Otsuka normal saline, Otsuka Pharmaceutical Factory, Tokushima, Japan) (50 μl) was added to the test tube, which was then placed at room temperature for 5 min. Then, 50 μl of the saline solution containing 50 nmol CPP and 10 nmol Tris(2-carboxyethyl)phosphine hydrochloride (Nacalai Tesque, Kyoto, Japan) was added to the solution (50 μl) in the test tube and stirred for 12 h at room temperature. In this procedure, lactosome formation and TPP encapsulation by the lactosome were expected. At the same time, maleimide-PSar_56_-PLLA_30_ in the lactosome is expected to react with CPP, which contains a cysteine residue at its C-terminus (Table 1). This lactosome mixture was diluted with 400 μl of saline, and passed through a 0.1 μm syringe filter (Membrane Solutions, Dallas, USA) to remove large aggregates. Low molecular weight molecules were excluded from the mixture using Amicon Ultra-0.5 (MWCO 50 kDa, Merck Millipore, Darmstadt, Germany). To analyze concentrations of CPP, TPP and ICG-PLLA_34_, absorption spectra of the lactosome complexes were measured using a BioSpec spectrometer (Shimadzu, Kyoto, Japan). The particle size, size distribution, polydispersity index (PDI), and zeta potential were measured by Zetasizer Nano ZSP (Malvern Instruments, Malvern, UK).

### 2.3. Analysis of lactosome complexes using size exclusion chromatography

Size exclusion chromatography was performed using a Superdex 200 10/300 GL column (GE Healthcare, Little Chalfont, UK) on an HPLC system (Shimadzu). The mobile phase was 10 mM Tris-HCl (pH 7.4) at a flow rate of 0.5 ml min^–1^. Absorbance was measured at 230, 280, 417, and 700 nm.

### 2.4. Detection of photogenerated singlet oxygen

Singlet oxygen (^1^O_2_) generation from the lactosome complexes (±TPP) was measured using Singlet Oxygen Sensor Green (SOSG) (Molecular Probes, Eugene, Oregon, USA). In a 96-well black plate with a clear bottom, 5 μl of the TPP-encapsulated lactosomes containing 1 μM TPP were mixed with 1 μl of 50 μM SOSG and diluted with 50 μl of distilled water. The lactosome solution was irradiated at 400–440 nm and 200 mW cm^–2^ for 50 s (10 J cm^–2^). Fluorescence intensity was measured at an excitation wavelength of 488 nm and an emission wavelength in the range 500–700 nm using a Jasco FP-6600 spectrofluorometer (JASCO, Tokyo, Japan).

### 2.5. Evaluation of cellular uptake of lactosome complexes containing CPPs

CHO cells were cultured at 37°C under 5% CO_2_ in Ham’s F12 medium (Wako Pure Chemical) supplemented with 10% fetal bovine serum (FBS, Nichirei Biosciences, Tokyo Japan), 100 units ml^–1^ penicillin (Gibco; Thermo Fisher Scientific, Waltham, Massachusetts, USA), and 100 μg ml^–1^ streptomycin (Gibco; Thermo Fisher Scientific). The cells were seeded at a density of 2 × 10^4^ cells/well in the 96-well plate, and incubated at 37°C under 5% CO_2_ overnight. The cells were then incubated at 37°C for 2 h with ICG-labeled lactosomes modified with CPPs (Tat, PTD4, DPV3, MPG^ΔNLS^, R9MPG, Pep1, or EB1) dissolved in 200 μl T buffer. The lactosome solution was exchanged for Ham’s F12 medium before the fluorescence imaging. The cellular fluorescence images were obtained by fluorescence microscopy using an Olympus IX71 microscope (Olympus, Tokyo, Japan) with a 40 × objective lens and a U-DM-CY7–3 mirror unit.

LysoTracker Green (Thermo Fisher Scientific) was used for investigating cellular localization of CPP-modified lactosomes. After incubation of the cells with CPP-modified ICG-labeled lactosomes, the lactosome solution was exchanged with Ham’s F12 medium containing 2 μM LysoTracker Green. The cells were further incubated for 3 h. Then, the cell medium was replaced with Ham’s F12 medium, and fluorescence images were obtained.

To evaluate the cellular uptake of the lactosome complexes, the ICG fluorescence images were obtained using an IVIS spectrum system (Xenogen, Hopkinton, Massachusetts USA) with a filter set specific for ICG (excitation at 745 nm and emission at 840 ± 10 nm). The ICG fluorescence intensity was estimated from the photon counts of the images. Then, the cell medium was replaced with solution containing 10 μl of Cell Counting Kit-8 solution (Dojindo, Kumamoto, Japan) and 90 μl of Ham’s F12 medium, and the cells were incubated for an additional 1.5 h. After incubation, the cell viability was measured by absorbance at 450 nm using the microplate reader SunriseR (Tecan Japan, Kanagawa, Japan). Lactosome uptake levels were calculated by dividing ICG fluorescence intensity by cell viability of each well.

### 2.6. Measurement of cell viability after treatment with the TPP-loaded lactosomes and light

NCI-N87 cells were cultured at 37°C under 5% CO_2_ in RPMI1640 medium (Wako Pure Chemical) supplemented with 10% FBS, 100 units ml^–1^ penicillin, and 100 μg ml^–1^ streptomycin. NCI-N87 cells were seeded at a density of 4 × 10^4^ cells/well in a 96-well plate, and incubated at 37°C for two days before treatment with the lactosome complex. CHO cells were cultured and seeded as described above. The effect of treatment with the TPP-loaded lactosomes and light on cell viability, which is related to PDT efficacy, was evaluated as follows: TPP-loaded lactosomes modified with CPPs (Pep1 or EB1) dissolved in 200 μl of T buffer (20 mM HEPES-KOH (pH 7.4), 115 mM NaCl, 5.4 mM KCl, 1.8 mM CaCl_2_, 0.8 mM MgCl_2_, and 13.8 mM glucose) were added to NCI-N87 cells (70% confluent) in the 96-well plate. The cells were incubated at 37°C for 1.5 h. After the lactosome solution was exchanged for T buffer, the cells were irradiated at 340–390 nm at 200 mW cm^–2^ for 100 s. After irradiation, the cells were further incubated for 24 h at 37°C under 5% CO_2_ in the medium. Then, the cell medium was exchanged for the solution containing 10 μl of Cell Counting Kit-8 solution and 90 μl of RPMI1640 medium, and the cells were incubated at 37°C for 2 h. The cell viability was measured by the absorbance at 450 nm using a microplate reader SunriseR. Statistical analysis was performed with EZR (Saitama Medical Center, Saitama, Japan) using one-way ANOVA followed by Dunnett’s test.

### 2.7. *Tumor*-*bearing mice and* in vivo *imaging*

Six-week-old male nude mice (BALB/c nu/nu) were purchased from Charles River. Five weeks before the imaging, PANC-1 cells (1 × 10^7^) suspended in 100 μl of 50% Matrigel Matrix (Corning Life Sciences, Corning, New York USA) in phosphate buffered saline was subcutaneously inoculated into the front left leg of mice. Two weeks after the first transplantation, NCI-N87 cells (4 × 10^6^) were suspended in the Matrigel solution and subcutaneously inoculated into the front right leg of mice. Three weeks after the second transplantation, CPP/TPP/ICG-lactosomes were injected into the tumor-bearing mouse via the tail vein. After 24 h, ICG fluorescence images were taken using the IVIS spectrum system (excitation at 745 nm and emission at 840 ± 10 nm, field of view = 12.5 cm (width and height), f/stop = 2, binning = 4, and exposure time at 3 s). During the imaging process, the mice were anesthetized using 3.0% isoflurane gas in oxygen flow (0.5 l min^–1^).

## 3. Results and discussion

### 3.1. Characterization of the CPP-modified lactosomes

**Figure 1.**
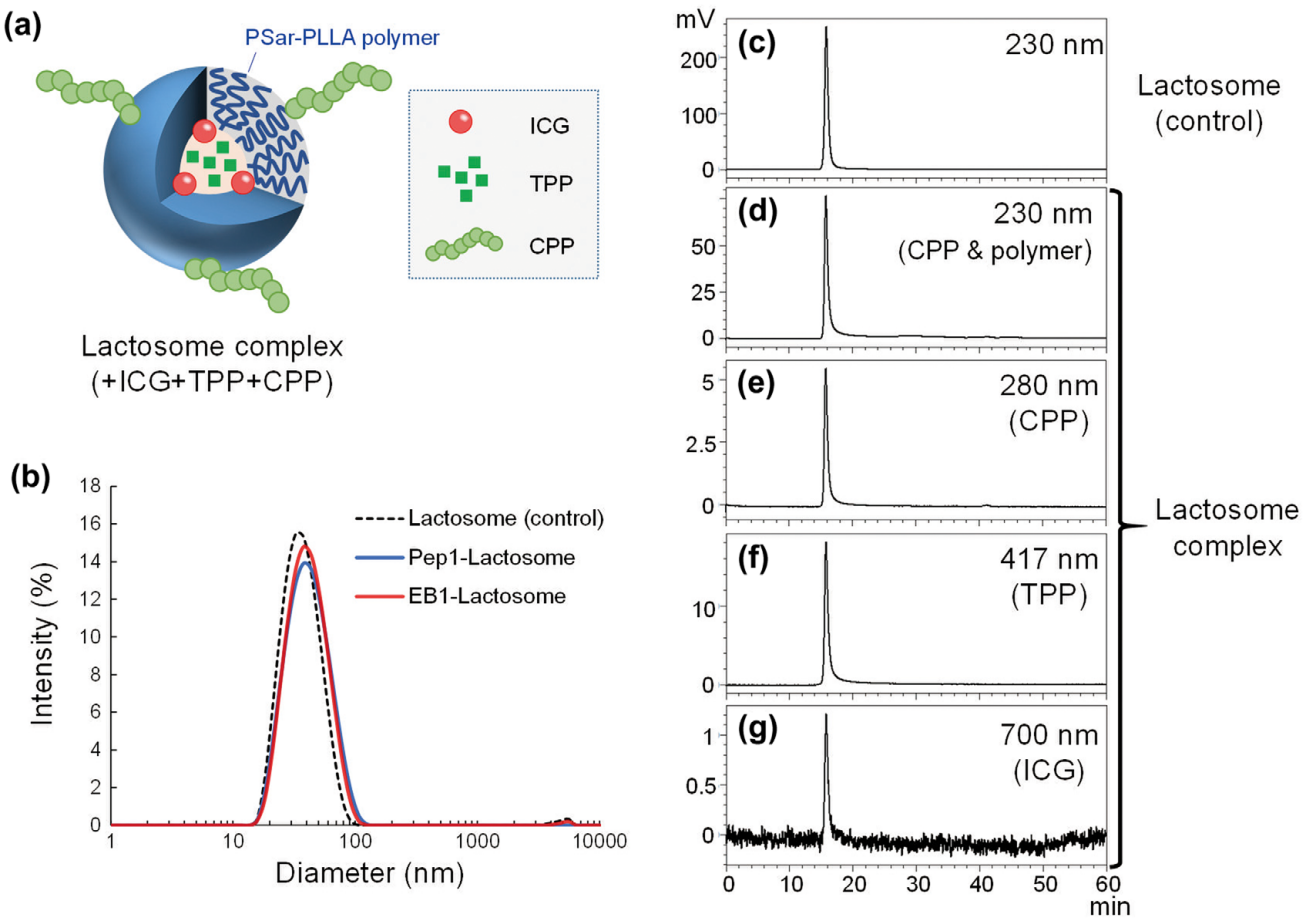
Characterization of lactosome complexes. (a) A diagram of the lactosome complex. (b) Size distribution of lactosomes modified with Pep1 or EB1 peptide. (c)–(g) Size exclusion chromatography of (c) unmodified and (d)–(g) EB1/TPP/ICG-lactosomes. Absorbance traces at (c), (d) 230 nm, (e) 280 nm, (f) 417 nm, and (g) 700 nm.

The particle sizes of lactosome complexes were measured by dynamic light scattering (DLS). The average particle sizes of the CPP-modified lactosomes were 36–49 nm, which were slightly larger than the unmodified lactosomes (34.2 nm) (Table 2, Figure 1(b)). The zeta potential value of CPP-modified lactosomes (e.g. Pep1-modified lactosome: 1.7 mV, EB1-modified lactosome: 1.6 mV) was higher than that of the unmodified lactosomes (–4.6 mV) (Table 2). The increase in the zeta potential seems to be due to positively charged CPPs. These results indicated that modification of lactosomes with CPPs increased their surface charge and particle size.

**Table 2.** Particle size, zeta potential and polydispersity index (PDI) of CPP-modified lactosomes.

CPP	Diameter (nm)	Zeta potential (mV)	PDI
-CPP	34.2±0.5	−4.6±0.2	0.13±0.01
Tat	36.2±0.3	−3.6±0.1	0.10±0.01
PTD4	37.0±0.4	−4.2±0.3	0.14±0.02
DPV3	37.0±0.4	−3.7±0.3	0.12±0.01
MPG^ΔNLS^	49.1±0.5	−2.9±0.6	0.29±0.02
R9MPG	43.9±1.5	−2.7±0.9	0.22±0.01
Pep1	37.7±0.6	1.7±0.1	0.13±0.02
EB1	38.3±0.2	1.6±0.3	0.12±0.01

These values were measured by DLS and laser Doppler electrophoresis (*n* = 3, mean ± standard deviation).

To confirm the incorporation of the CPP (EB1), TPP, and indocyanine green (ICG)-PLLA into the lactosomes, size exclusion chromatography was performed using a Superdex 200 10/300 GL column and the HPLC system (Figure 1(c)–(g)). The purified lactosome complex containing EB1, TPP, and ICG (EB1/TPP/ICG-lactosome) was eluted as a single peak from the column, and its retention time was the same as in the unmodified lactosomes (Figure 1(c) and 1(d)). Absorbance traces of the chromatography at 230, 280, 417, and 700 nm suggested that EB1, TPP, and ICG-PLLA were successfully incorporated into lactosome complexes (Figure 1(c)–(g)). EB1 is thought to bind to maleimide-PSar_56_-PLLA_30_ in the lactosome, and TPP is thought to be encapsulated in the hydrophobic core of the lactosome by hydrophobic interaction.

### 3.2. Cellular uptake of CPP-modified lactosomes

**Figure 2.**
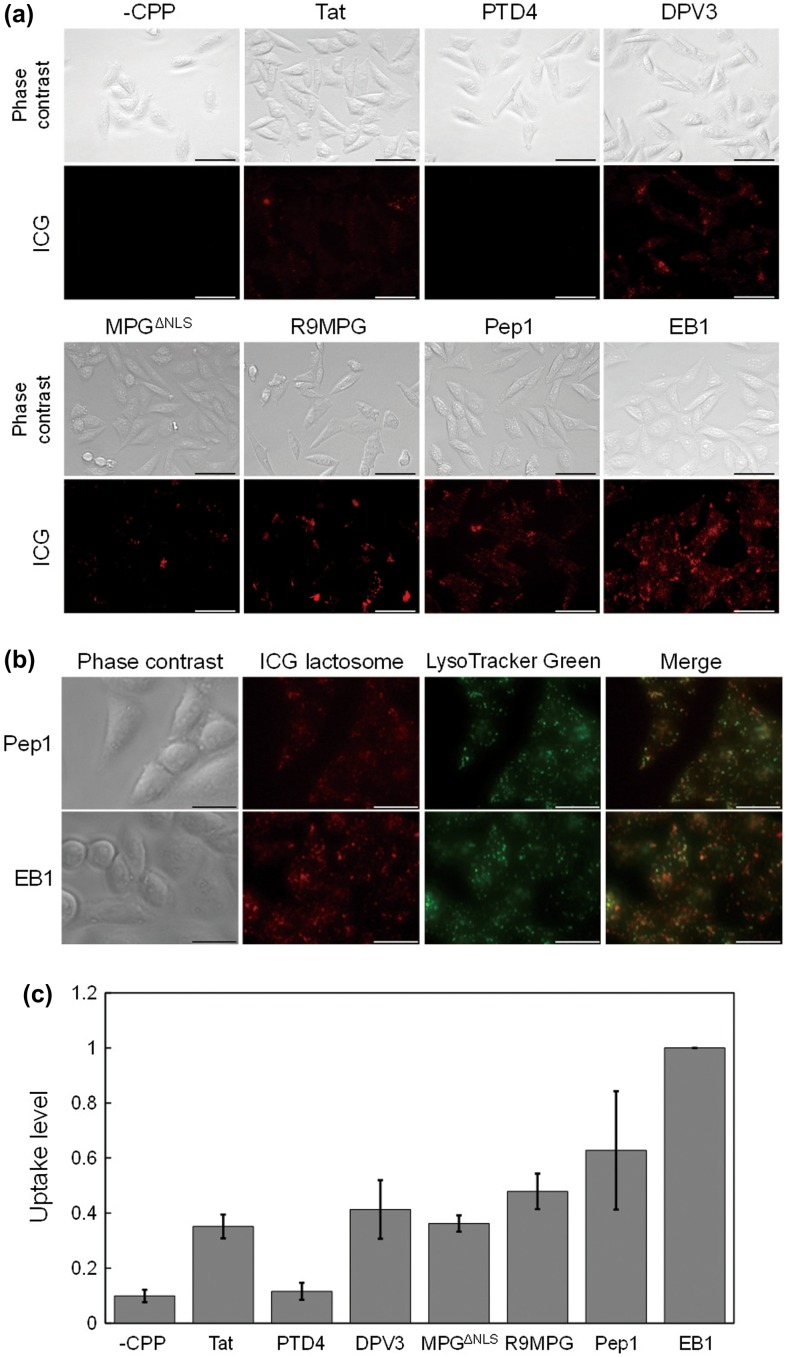
Cellular uptake of the ICG-labeled lactosomes modified with CPPs (Tat, PTD4, DPV3, MPG^ΔNLS^, R9MPG, Pep1, or EB1). CHO cells were cultured with 10 nmol of the lactosome complexes for 2 h at 37°C. (a) Optical microscopy images of the cells cultured with the ICG-labeled and CPP-modified lactosomes. Scale bars represent 50 μm. (b) Images of Pep1- and EB1-modified ICG-lactosomes in the cells co-stained with LysoTracker Green. Scale bars indicate 20 μm. (c) Cellular uptake level calculated by dividing ICG fluorescence intensity by cell viability relative to that of the EB1-modified lactosomes. Values are expressed as mean ± standard error of the mean, *n* = 4.

Internalization of CPP-modified lactosomes by CHO cells was observed by fluorescence microscopy (Figure 2(a)). ICG-labeled CPP-modified lactosomes were added to CHO cells and incubated at 37°C for 2 h. After the incubation and washing steps, significantly strong ICG fluorescence was observed in the CHO cells treated with the Pep1- and EB1-modified lactosomes, compared to the lactosomes without CPP. The CPP-modified lactosomes showed dotted localization. Pep1- and EB1-modified lactosomes co-localized with LysoTracker Green (Figure 2(b)), indicating that they localized mainly in acidic organelles, including endosomes and lysosomes. Furthermore, the lactosome uptake level of the cells was estimated from the ICG fluorescence intensity and cell viability of each culture well (Figure 2(c)). The EB1-modified lactosomes was the most abundantly internalized by the cells. Pep1-modified lactosomes were also highly internalized (their uptake level relative to the EB1-modified lactosomes was 0.62). These results indicated that cellular uptake of lactosomes was improved by the addition of CPPs, especially EB1 and Pep1. These results showed that cellular uptake efficiency of the lactosomes modified with amphipathic CPPs was higher than that of lactosomes modified with non-amphipathic CPPs, which may be related to the binding efficiency of the CPPs to lactosomes. There was no relationship between the size of the CPP-modified lactosomes (Table 2) and the cellular uptake efficiency. Zeta potential seems to be related to the cellular uptake efficiency because both EB1- and Pep1-modified lactosomes had positive zeta potential (Table 2), while the others had negative potential. This effect seems logical since the first step in CPP uptake is usually interaction with negative surface of the plasma membrane.

### 3.3. Singlet oxygen generation from TPP-loaded lactosomes

**Figure 3.**
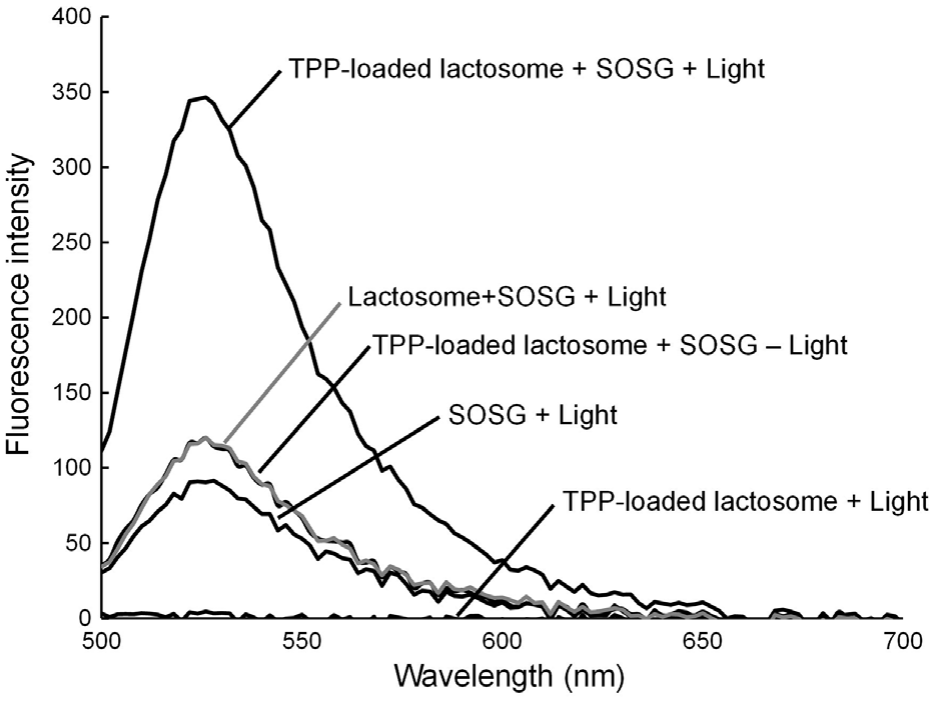
Fluorescence spectra of the Singlet Oxygen Sensor Green (SOSG) with TPP-loaded or unloaded lactosomes after photoirradiation at 400–440 nm at 200 mW cm^–2^ for 50 s. The excitation wavelength was 488 nm.

PDT with the use of a photosensitizer is generally based on photoinduced singlet oxygen generation from the photosensitizer. TPP encapsulated in lactosomes may lose its photosensitizing ability. Thus, we confirmed singlet oxygen generation from TPP-loaded lactosomes using the singlet oxygen indicator SOSG. Figure 3 indicates that singlet oxygen was photo-dependently generated from TPP-loaded lactosomes. Therefore, this lactosome complex can be applied for PDT.

### 3.4. Photoinduced cell killing efficacy of the CPP-modified TPP-loaded lactosomes

**Figure 4.**
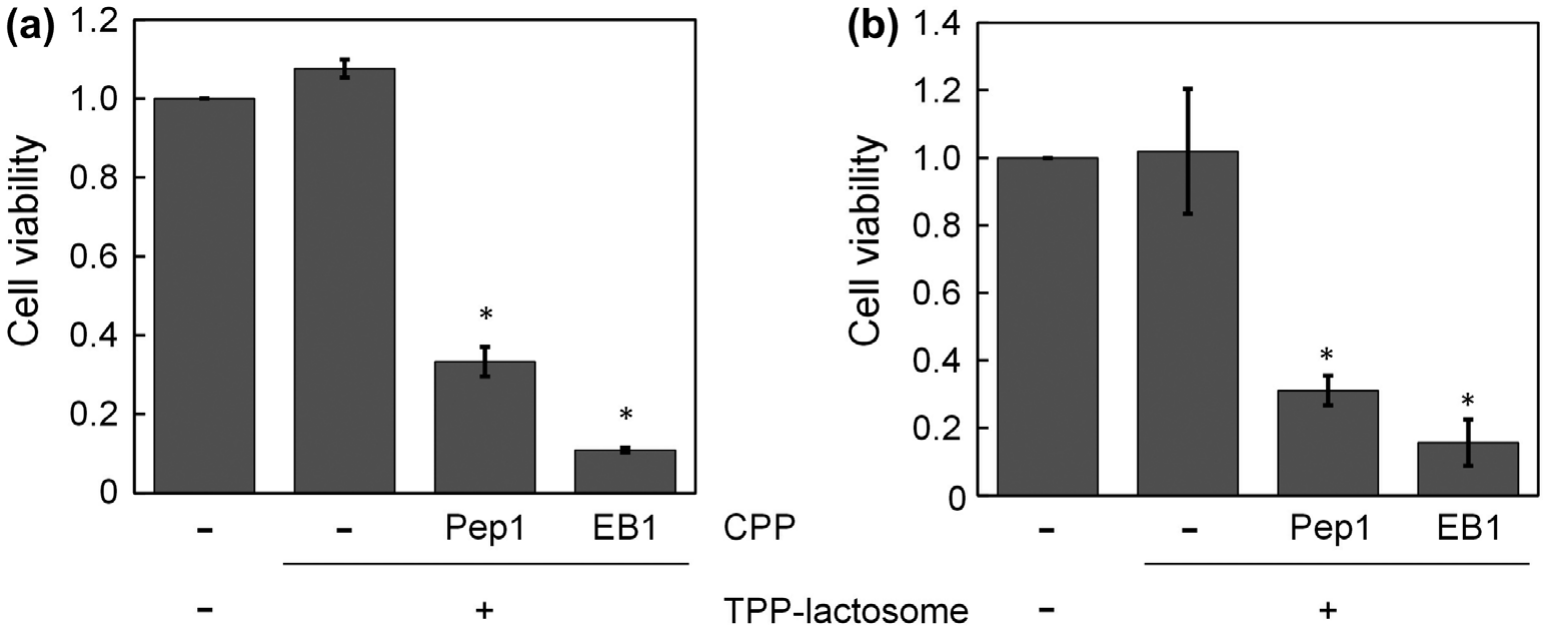
Efficacy of the PDT treatment in (a) NCI-N87 and (b) CHO cells. After exposing the cells to CPP/TPP-lactosomes and light at wavelengths of 340–390 nm at 200 mW cm^–2^ for 100 s, cell viability was measured using the Cell Counting Kit-8. Cell viability values were normalized to that of untreated cells (- CPP, - TPP-lactosome), which were irradiated under conditions similar to the cells treated with CPP-lactosomes. Values are expressed as the mean ± standard error of the mean, *n* = 3. *p < 0.001 versus the untreated cells.

Cell treatment with a photosensitizer and light is called PDT treatment and can be used for killing malignant or cancer cells. To evaluate the effect of PDT treatment using the CPP-modified and TPP-loaded lactosomes (CPP/TPP-lactosomes), cell viability was measured after treatment with CPP/TPP-lactosomes followed by photoirradiation, and 24 h culture in the medium. EB1/TPP-lactosomes and Pep1/TPP-lactosomes with photoirradiation efficiently induced the killing of NCI-N87 cells (89% and 67%, respectively, compared to the experiment without CPP/TPP-lactosomes) (Figure 4(a)). Similar results were obtained in CHO cells (Figure 4(b)). The cell damage observed with these CPP/TPP-lactosomes is thought to be due to the photo-generated singlet oxygen (Figure 3). The efficiency of the cell killing was related to the cellular uptake efficiency of CPP-modified lactosomes (Figure 2) (EB1 > Pep1 > -CPP). These results indicated that the EB1 peptide was the most effective CPP for cellular internalization of TPP lactosomes.

### 3.5. In vivo *imaging*

**Figure 5.**
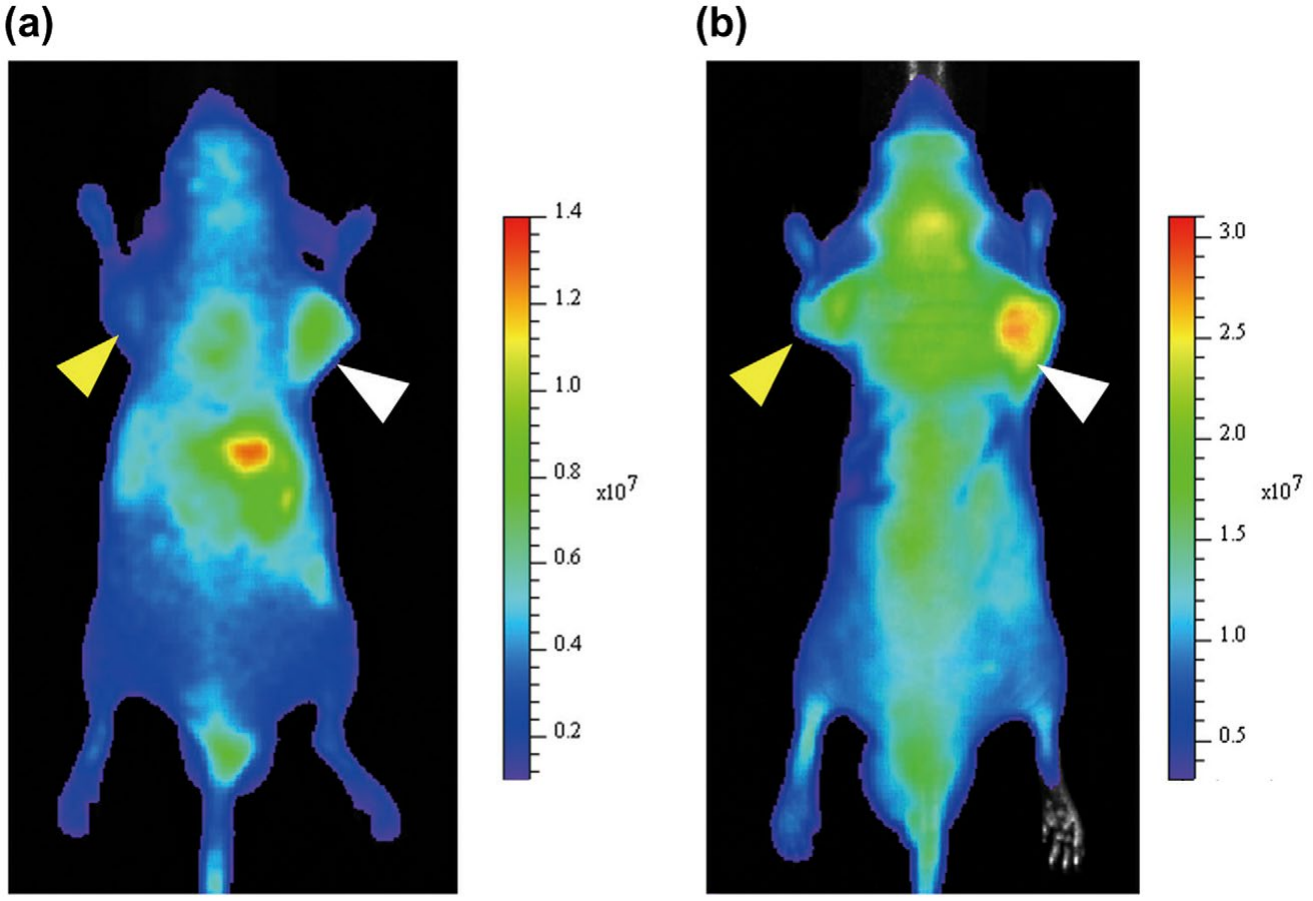
*In vivo* imaging of EB1/TPP/ICG-lactosomes (a) and TPP/ICG-lactosome (b). Ten-week-old male BALB/c nu/nu mice, grafting NCI-N87 and PANC-1 at front legs (right; NCI-N87, left; PANC-1) were used. The ICG image was obtained 24 h after administration of lactosomes to the tumor-bearing mouse. Arrows indicate tumor sites (white; NCI-N87, and yellow; PANC-1). The color bar indicates the fluorescence radiant efficiency (photons s^−1^ cm^−2^ steradian^−1^).

To confirm the accumulation of EB1/TPP/ICG-lactosomes in tumor sites, lactosomes were administrated to mice bearing NCI-N87 and PANC-1 tumors, and ICG fluorescence images were obtained. Reproducibility was confirmed using three mice, and the representative image is shown in Figure 5. Twenty-four h after injection, ICG fluorescence was detected in the NCI-N87 tumor, indicating that the EB1-modified lactosome accumulated in NCI-N87 tumors and liver (Figure 5(a)), though it accumulated in NCI-N87 tumors less than the lactosome lacking the CPP (Figure 5(b)). This accumulation in liver indicates that the escape ability (stealth property) of EB1-modified lactosome from RES is slightly lower than the lactosome lacking the CPP. In contrast to the NCI-N87 tumor, only slight ICG fluorescence was detected in the PANC-1 tumor (Figure 5(a)). These observations are consistent with those of the previous reports.[28,29] The accumulation of lactosomes in NCI-N87 tumors has also been reported in a study that used HER2-modified AB-type lactosomes.[28] Pancreatic tumors are known to be poorly permeable, and among polymeric micelles with diameters of 30, 50, 70, and 100 nm, only the 30 nm micelles accumulated in pancreatic tumors.[29] Since the size of the EB1-modified lactosomes used in this study was ~38 nm, reducing the size of the CPP-modified lactosomes may increase their accumulation in tumors.

## 4. Conclusions

The A_3_B-type lactosome, a biocompatible and biodegradable polymeric nanomicelle, was modified with CPPs to improve its cellular uptake. Among the seven kinds of CPPs (Tat, PTD4, DPV3, MPG^ΔNLS^, R9MPG, Pep1, and EB1), amphipathic EB1 and Pep1 peptides greatly improved the uptake efficiency of the lactosomes. The CPP-modified lactosomes internalized by cells were localized mainly in endosomes or acidic organelles. We also conducted PDT experiments using the CPP-modified and photosensitizer-loaded lactosomes. Cell killing was efficiently photoinduced using the EB1/TPP and Pep1/TPP lactosomes. *In vivo* imaging of the EB1/TPP/ICG-lactosomes showed that they accumulated in NCI-N87 tumors in mice. More efficient tumor accumulation may be accomplished through the size-control of the CPP-modified lactosomes. The CPP-modified lactosome is promising as an efficient drug carrier. This study demonstrated that CPP-modified lactosomes encapsulated the hydrophobic agent TPP and delivered it into cells. Thus, CPP-modified lactosomes can deliver hydrophobic agents into cells. These lactosomes may also be able to deliver hydrophilic drugs such as proteins and nucleic acids into cells by attaching hydrophobic modifications to them.

## Funding

This work was supported by the Project for Development of Innovative Research on Cancer Therapeutics (P-DERECT) and Grants-in-Aid for Scientific Research on Innovative Areas “Nanomedicine Molecular Science” to T. O. [26107711].

## Disclosure statement

No potential conflict of interest was reported by the authors.
